# Ethanol-Induced Hepatotoxicity and Alcohol Metabolism Regulation by GABA-Enriched Fermented *Smilax china* Root Extract in Rats

**DOI:** 10.3390/foods10102381

**Published:** 2021-10-08

**Authors:** Naila Boby, Eon-Bee Lee, Muhammad Aleem Abbas, Na-Hye Park, Sam-Pin Lee, Md. Sekendar Ali, Seung-Jin Lee, Seung-Chun Park

**Affiliations:** 1 Laboratory of Veterinary Pharmacokinetics and Pharmacodynamics, College of Veterinary Medicine, Kyungpook National University, Daegu 41566, Korea; nailaboby@knu.ac.kr (N.B.); eonbee@gmail.com (E.-B.L.); syedaleemabbas77@gmail.com (M.A.A.); alipharm2000@gmail.com (M.S.A.); 2 Laboratory Animal Centre, Daegu Gyeongbuk Medical Innovation Foundation, Daegu 41061, Korea; skyphn@naver.com; 3 Department of Food Science and Technology, Keimyung University, Daegu 42601, Korea; splee@knu.ac.kr; 4 Department of Biomedical Science and Department of Pharmacology, School of Medicine, Brain Science and Engineering Institute, Kyungpook National University, Daegu 41944, Korea; 5 Department of Pharmacy, International Islamic University Chittagong, Kumira, Chittagong 4318, Bangladesh; 6 Developmental and Reproductive Toxicology Research Group, Korea Institute of Toxicology, Daejeon 34114, Korea

**Keywords:** *Smilax china*, alcohol metabolism, superoxide dismutase, alcohol dehydrogenase, aldehyde dehydrogenase

## Abstract

Chronic alcohol consumption can cause hepatic injury and alcohol-induced toxicities. Extracts from *Smilax china* root have been widely used in traditional medicine and for their potential pharmacological benefits. We aimed to determine if fermented *Smilax china* extract (FSC) regulates alcoholic fatty liver and liver injury using two in vivo experiments. Sprague-Dawley rats were administered ethanol (3 g/kg b.w.; po) with or without FSC pretreatment to induce an acute hangover. In another experiment, rats were fed either a normal or Lieber-DeCarli ethanol (6.7%) diet with or without FSC pretreatment (125, 250, and 500 mg/kg b.w.; po) for 28 days. Serum biomarkers, liver histopathology, and the mRNA levels of anti-inflammatory, antioxidant, lipogenic, and lipolytic genes were analyzed. FSC pretreatment significantly reduced blood alcohol and acetaldehyde concentrations, upregulated the mRNA expression of alcohol dehydrogenase, aldehyde dehydrogenase, and superoxide dismutase, and decreased the activities of liver enzymes in a dose-dependent manner. It also downregulated SERBP-1c and upregulated PPAR-α and reduced the gene expression of the anti-inflammatory cytokine IL-6 in the liver. The final extract after fermentation had increased GABA content. Furthermore, FSC was found to be safe with no acute oral toxicity in female rats. Thus, FSC increases alcohol metabolism and exhibits antioxidant and anti-inflammatory effects to induce hepatoprotection against alcohol-induced damage. It may be used as a functional food ingredient after excess alcohol consumption.

## 1. Introduction

The liver plays a pivotal role in regulating physiological processes and performs various vital activities such as metabolism, secretion, and storage. Moreover, the liver is responsible for the detoxification of xenobiotics and drugs. Therefore, the development of liver diseases can result in serious health issues. These health issues are classified as degenerative disorders (cirrhosis), acute or chronic liver inflammatory diseases (hepatitis), or non-inflammatory diseases (hepatosis) [1]. Liver disorders can arise as a direct result of infection, autoimmune disorders, excessive consumption of alcohol, or consumption of toxic substances such as peroxidized oil, drugs, antibiotics, chlorinated hydrocarbons, and carbon tetrachloride. Alcoholism and alcohol abuse are a common global problem that affects all cultures. Alcoholic liver disease (ALD) is a major cause of chronic liver damage caused by excessive alcohol consumption that causes deaths worldwide [2]. A hangover that occurs the next morning following an alcohol overdose results in temporary physical and psychological problems such as headache, sweating, gastrointestinal complaints, and fatigue. These adverse effects are due to a combination of main metabolic products of alcohol and acetaldehyde [3,4]. Increased reactive oxygen species (ROS), produced as a result of alcohol metabolism, drives alcohol-induced liver damage due to impaired antioxidant activity in the liver [5,6].

Although several allopathic drugs have been developed, the search for an effective therapeutic agent for hepatotoxicity is ongoing. Plant-based formulations are routinely used for treating liver diseases in traditional medicine systems, but only a few of these formulations have been evaluated for their pharmacological efficacy [7]. Plants rich in flavonoids, high-efficiency antioxidants, and radical scavengers can prevent liver damage [8]. *Smilax china*, a member of the Smilacaceae family, is native to East Asia (e.g., Korea, China, Japan, and Taiwan) [9]. It is used in traditional Chinese herbal medicine and contains saponins, glucosides, gum starch, flavonoids, tannins, and alkaloids [10]. Feng et al. found dihydrokaempferol (1), 3,5,4/-trihydroxystilbene (2), 3,5,2/,4/-tetrahydroxy-stilbene (3), dihydrokaempferol 3-O-α-L-rhamnoside (engeletin, 4), and quercetin 4/-O-β-D-glucoside as phenolic compounds isolated from the roots of *S. china* [11]. Shao et al. identified dihydrokaempferol-5-O-β-D-glucoside (I), engeletin (II), isoengeletin (III), dihydroquercetin-3-O-glycoside (IV), 3, 5, 7, 3/, 5/-pentahydroxy-flavanonol (V), astilbin (VI), quercetin-3/-O-glycoside (VII), piceid (VIII), scirpusin A (IX), resveratrol (X), and oxyresveratrol (XI) as flavonoids and stilbenes [12]. *S. china* is reported to mainly contain flavones, isoflavones, taxifolin-3-O-glycoside, piceid, oxyresveratrol, engeletin, resveratrol, and scirpusin [13]. The stems and roots of *S. china* are mainly used as food but are also used as traditional medicine for detoxification, nephritis, heavy metal poisoning, and rheumatoid arthritis [14]. Recently, *S. china* was reported to counter oxidative stress [15], cancer [14], heavy metal poisoning [16], microbial infections [17], and obesity [16]. Solomon et al. demonstrated its hepatoprotective effect in a model of carbon tetrachloride-induced alcohol damage [1]. The effect of fermented *S. china* extract (FSC) on alcohol-induced liver injury is not reported. The fermented extract is known to enhance the activities of catalase (CAT) and superoxide dismutase (SOD) [2]. Fermented plant extracts have been investigated as functional foods and nutraceuticals. We believe that FSC can be used as a functional food and nutraceutical in the management of ALD. In this study, we investigated the biochemical, genetic, and histopathological changes after FSC administration in rats with alcohol-induced liver damage. We found that FSC improves ALD by lowering the serum levels of acetaldehyde, improving the biochemical markers of liver disease, increasing the gene expression of alcohol-metabolizing hepatic enzymes, and alleviating the lesions in the liver.

## 2. Materials and Methods

### 2.1. Extract Preparation and Analysis

#### 2.1.1. Preparation of FSC

The dried and crushed roots of *S. china* were macerated with water (1:10) for 8 h at 100 °C. *S.* *china* extract (50 mL) was co-fermented with *Bacillus subtilis* HA (KCCM 10775P) and *Lactobacillus plantarum* EJ2014 (KCCM 11545P) to produce a novel functional food ingredient enriched with γ-aminobutyric acid (GABA). Briefly, the *S. china* extract was mixed with 3% glucose and 5% monosodium L-glutamate (MSG) and autoclaved for 15 min at 121 °C. Then, *Bacillus subtilis* HA (KCCM 10775P) starter culture was inoculated and incubated at 42 °C for 3 days. After performing the first fermentation, skim milk (1% v/v) and glucose (1.5% v/v) solutions were added. *Lactobacillus plantarum* EJ2014 (KCCM 11545P) was inoculated and incubated at 30 °C for 7 days for secondary fermentation, and the final fermentation product of *S. china* was lyophilized in a freeze dryer at −70 °C for 3 days (Freeze Dryer, Ilshin BioBase Ltd., Ede, Netherlands; Pilot LP20) [2]. *Hovenia dulcis* fruit extract (HDE) was purchased from ES Food Ingredients Co., Ltd. (Gunpo, Korea).

#### 2.1.2. Quantification of GABA Content

The amino acid content of FSC was quantified using an L-8800 amino acid auto-analyzer (Hitachi Ltd., Tokyo, Japan) according to the manufacturer’s instructions. All extracts were lyophilized and subsequently used. In summary, 0.1 g of fermented extract was mixed with 5% trichloroacetic acid (TCA) solution and vortexed. Then, the mixture was passed through a 0.45 µm cellulose acetate filter. The filtrate was analyzed with a column packed with a Hitachi custom ion exchange resin (4.6 mm ID’ 60 mm L), and the mobile phases comprised a buffer, physiological fluid assay buffer (PF)-1, 2, 3, 4, PF-RG (PF-regenerating solution), R-3, and C-1. The detection wavelength and flow rate of the buffer were 570 nm and 0.55 mL/min, respectively. The temperatures of the column and reactor were set to 50 °C and 135 °C, respectively. The injection volume for all samples and standard solutions was 20 µL.

### 2.2. Animals and Experimental Design

#### 2.2.1. Animals

Male Sprague-Dawley rats (body weight [b.w.], 220 g) were obtained from Jung-Ang Animal Laboratory (Seoul, Korea). The rats were housed in a controlled environment (22 ± 2 °C, 50 ± 10% relative humidity, 12 h light/dark cycle) and fed a standard laboratory chow (ad libitum) diet and water. The Animal Care and Use Committee of Kyungpook National University, Daegu, Korea (KNU 2017-50) approved the protocols for the animal studies. Rats were acclimatized to laboratory conditions for 7 days prior to the commencement of the experiment. The total number of rats used in the study (n = 36 and n = 36) was calculated by the G*power program based on the number of groups (6), α-error probability (0.05), power (1-β error probability) (0.8), and effect size (0.5) [18,19].

#### 2.2.2. Experimental Design for Alcohol Metabolism after Acute Ethanol Administration

**Figure 1:**
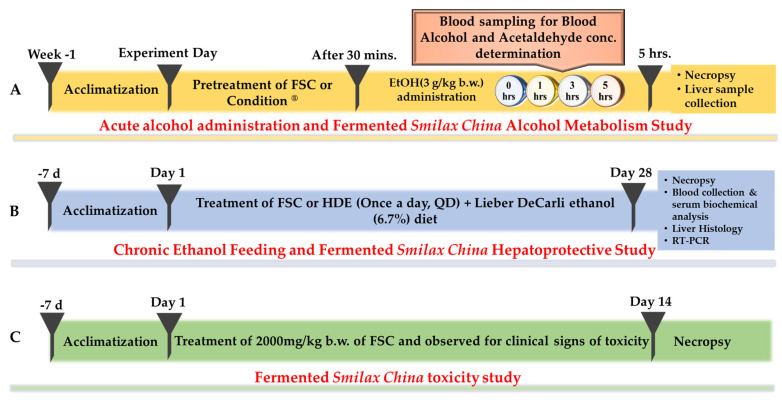
In vivo experimental design: (**A**) The rats were pretreated with different doses of fermented *Smilax china* extract (FSC) prior to the oral administration of ethanol (3 g/kg b.w.). Then, blood samples were collected at different time intervals (0, 1, 3, and 5 h), and alcohol metabolism-associated effects of FSC were studied. (**B**) The different groups of rats were fed Lieber-DeCarli ethanol (6.7%) diet with or without FSC pretreatment (once daily) for 28 days. After sacrifice, the hepatoprotective effects of FSC were studied. (**C**) The acute toxicity of FSC was studied; bolus 2000 mg/kg b.w. of FSC was administered to female rats, and the rats were observed over 14 days.

To evaluate the effects of FSC on alcohol metabolism, rats (n = 36) were randomly divided into six groups (n = 6/group). Group (ND)—normal diet; Group (NC)—ethanol only (negative control); Group (PC)—Condition^®^ (positive control) + ethanol; Group (FSC 125)—125 mg/kg b.w. of FSC + ethanol; Group (FSC 250)—250 mg/kg b.w. of FSC + ethanol; Group (FSC 500)—500 mg/kg b.w. of FSC + ethanol. Water, Condition^®^, or FSC was administered to each group via the intragastric route, and 30 min later, ethanol (3 g/kg b.w.) was administered. Blood samples were collected at different time intervals (0, 1, 3, and 5 h), as shown in Figure 1A [20]. Condition^®^, used in the present study as a positive control, is a commercial hangover removal solution (CJ Corp., Korea). It is composed of glutamate and alcohol dehydrogenase (ADH-1) and has been recommended before alcohol consumption (200 mL/70 kg b.w.) [21].

#### 2.2.3. Experimental Design

To evaluate the hepatoprotective effect of FSC against chronic alcohol-induced liver damage, rats (n = 36) were randomly divided into six groups (n = 6 rats/group) and treated for 28 days with FSC once a day (QD) with or without Lieber-DeCarli ethanol (6.7%) diet (Figure 1B) [22,23]. Group (ND)—Lieber-DeCarli liquid control diet (DYET# 710027, Dyets. Inc. USA) (isocalorically substituted maltose dextrin for ethanol over the entire feeding period); Group (NC)—free access to Lieber-DeCarli ethanol (6.7%) diet (DYET# 710260, Dyets. Inc. USA) (negative control); Group (PC)—250 mg/kg b.w. of HDE (QD) + Lieber-DeCarli ethanol diet (positive control); Group (FSC 125)—125 mg/kg b.w. of FSC (QD) + Lieber-DeCarli ethanol diet; Group (FSC 250)—250 mg/kg b.w. FSC (QD) + Lieber-DeCarli ethanol diet; Group (FSC 500)—500 mg/kg b.w. FSC (QD) + Lieber-DeCarli ethanol diet. HDE (*Hovenia dulcis*) was used as a positive hepatoprotective agent in alcohol-induced hepatotoxic rats. HDE is approved as a dietary supplement to prevent alcohol-induced liver damage by the Ministry of Food and Drug safety (MFDS) in Korea [2,24].

#### 2.2.4. Alcohol and Acetaldehyde Concentration in Serum

Blood alcohol and acetaldehyde concentrations were measured at 0, 1, 3, and 5 h following the oral administration of 3 g/kg b.w. of ethanol to rats. Blood samples were obtained from the tail vein and collected in microcentrifuge tubes. Then, blood samples were centrifuged at 1000 ×g for 15 min at 4 °C, and serum was separated and stored at −70 °C until further analysis. Alcohol concentrations in the blood serum were determined following manufacturer instructions by enzyme-based colorimetric assay kits (Catalog # ab65343, Abcam, Cambridge, MA, USA), and the absorbance was measured at 570 nm spectrophotometrically using a spectrophotometer (EPOCH™-2, BioTek instruments, Seoul, Korea). Meanwhile, aldehyde concentration determination was performed using an aldehyde quantification assay kit (Catalog # ab112113, Abcam, UK), and the absorbance was assessed at 550 nm spectrophotometrically using a spectrophotometer (EPOCH™-2, BioTek instruments, Seoul, Korea). Reaction mix without sample was used as a blank control.

#### 2.2.5. Measurement of Liver Biomarkers in Serum

The protective effects of FSC from ethanol-induced liver damage were determined by measuring the activities of liver biomarkers in serum samples. Briefly, the blood samples were collected from the heart of rats in microcentrifuge tubes. After collection, the blood was centrifuged at 1000 ×g for 15 min at 4 °C, and serum was collected immediately. Serum concentrations of alkaline phosphatase (ALP), aspartate aminotransferase (AST), alanine aminotransferase (ALT), and albumin (ALB) were analyzed using an auto serum analyzer to determine the liver function (Thermo Electron, Santa Cruz, CA, USA). Each sample was assayed thrice.

#### 2.2.6. Histopathology

Liver tissue was isolated, and a small portion was fixed with 10% formalin, dehydrated in graduated ethanol (502013;100%), cleared in xylene, and then embedded in paraffin. Sections of thickness 4–5 µm were prepared and then stained with hematoxylin and eosin (HE) dye. These were examined for histopathological changes under a microscope (DMIRE2, Leica, Wetzlar, Germany). The histological scoring was based on the appearance of major pathologic changes such as degrees of hepatic necrosis, inflammation, balloon degeneration, and fatty degeneration, according to the report by Yang et al. [25]. The scores were 0, 1, 2, 3, or 4 (with 0 being no lesion noted and 4 indicating the most severe lesions). According to the importance of pathological changes, scores were multiplied using weighting factors. The remaining portion of the liver was frozen in liquid nitrogen and stored at −80 °C for further analysis.

#### 2.2.7. Total RNA Extraction and Quantitative Real-Time PCR (qRT-PCR)

A total of 100 mg of liver tissue was washed with cold phosphate-buffered saline and homogenized in 1 mL of TRIzol reagent (Invitrogen, Carlsbad, CA, USA) using a homogenizer (T-10 basic Ultra-Turrax, IKA Werke GmbH & Co. KG, Germany) on ice. Total RNA was extracted using the TRIzol reagent according to the manufacturer’s protocol. For cDNA generation, 1 mg of RNA was subjected to reverse transcription using the cDNA EcoDry Premix (Takara, Tokyo, Japan) according to the manufacturer’s protocol. The cDNA product was amplified by qRT-PCR using specific primers, with ß-actin as the internal reference [25]. In brief, 1 mg of cDNA was added to 2X iQ SYBR Green Supermix (Bio-Rad, Hercules, CA, USA) containing 1 pM of each primer. PCR amplification was conducted in a Bio-Rad Real-time thermal cycler CFX96 (Bio-Rad, Hercules, CA, USA) with the following cycling profile: denaturation at 95 °C for 15 min, followed by 40 cycles of denaturation at 95 °C for 20 s, annealing at 55 °C to 60 °C for 20 s, and then elongation at 70 °C for 30 s. The gene expression levels were determined using the comparative cycle threshold method and are shown as 2^−^^△△Ct^ by using the Gene Expression Analysis for CFX manager v1.6 Real-Time PCR Detection System (Bio-Rad, Hercules, CA, USA). Finally, the specificity of each PCR product was analyzed from the melting peaks.

### 2.3. Acute Oral Toxicity Test

Eight-week-old female Sprague-Dawley rats (n = 10) were obtained from Orient Bio Inc. (Gyeonggi-do, Korea). These animals were housed in the same manner as detailed in the “Animal experimental design” section. The acute oral toxicity test was performed with slight modifications to a previously reported method. Rats were randomly assigned to control and test groups (n = 5/group). A single dose of FSC (2000 mg/kg b.w.) was administered via the intragastric route to the animals according to OECD test guideline 423 [26]. A standard pellet diet (Hyochang Science, Daegu, Korea) and distilled water were provided ad libitum. The animals were under constant observation for abnormal signs and symptoms for the first 12 h after FSC administration. They were further observed once a day for 2 weeks. The body weight of each animal was recorded prior to FSC administration, and changes to body weight and feed consumption weight were measured twice per week for 14 days after treatment (Figure 1C). Animals were sacrificed after the experimental period, and major organs were collected and inspected for gross lesions.

### 2.4. Statistical Analysis

All data are presented and shown as the mean ± standard error of the mean (SEM) for each group. One-way analysis of variance (ANOVA) followed by Tukey’s multiple comparison test was used to calculate the statistical difference. A value of *p* < 0.05 was considered to indicate a statistical significance. For the acute oral toxicity study, a *t*-test was used. Statistical analysis was conducted using GraphPad Prism version 7.0 software (GraphPad Software, CA, USA).

## 3. Results

### 3.1. Fermentation Effects on GABA Content

**Figure 2:**
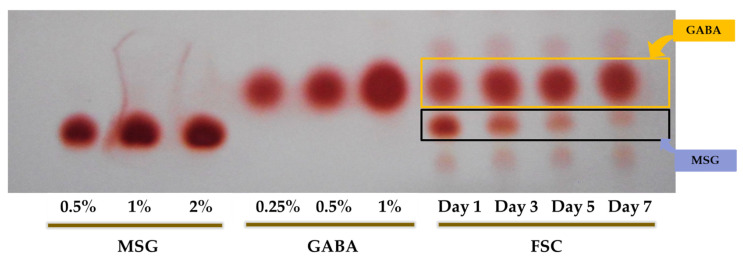
Fermentation effects on GABA production patterns in FSC: Thin-layer chromatogram for MSG standard (0.52013;2%), GABA standard (0.232013;1%), and two-fold dilution of FSC samples collected at different time intervals (1, 3, 5, and 7 days) during fermentation.

The GABA content of *S. china* extract fermented by *B. subtilis* HA and *L. plantarum* EJ2014 was qualitatively determined using thin-layer chromatography (TLC) (Figure 2). The level of MSG released in the medium gradually reduced during fermentation, and the level of GABA in FSC rapidly increased. After 1 d of fermentation, the GABA content in FSC was 0.5%, which then increased slowly to ~ 1% on day 7. This finding indicated that fermentation with *B. subtilis* HA and *L. plantarum* EJ2014 enhanced the GABA content in FSC. Subsequent experiments in this study used the FSC after 7 d fermentation followed by lyophilization.

### 3.2. Effect of FSC on Alcohol Metabolism

#### 3.2.1. Effect of FSC on Blood Concentration of Alcohol and Acetaldehyde

**Figure 3:**
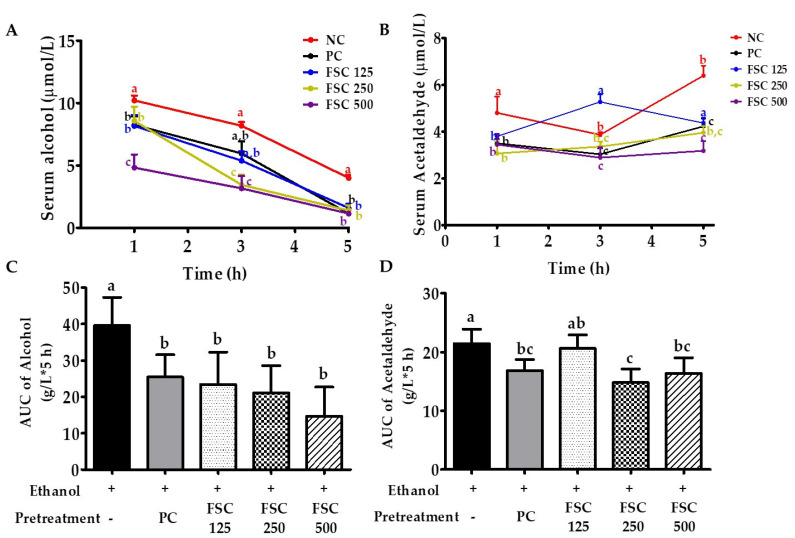
The effect of FSC on blood concentration and AUC of alcohol (**A**,**C**) and acetaldehyde (**B**,**D**) in alcohol-loaded rats before and after acute alcohol consumption. Briefly, rats were orally administrated water, Condition^®^, or FSC. Then, after 30 min, a dose of ethanol (3 g/kg) was administered. Blood was collected from the tail vein at 1, 3, and 5 h after ethanol administration. Group NC, oral administration of 3 g/kg b.w. of alcohol only; Group PC, pretreated with Condition^®^; Group FSC 125, FSC 250, and FSC 500, pretreated with FSC at 125, 250, and 500 mg/kg b.w., respectively. Data represent the mean ± SEM (n = 6). Bars with different letters show significant differences between groups (*p* < 0.05) determined by analysis of variance and Tukey’s multiple comparison test.

Blood alcohol concentration (BAC) peaked 1 h after ethanol administration (10.2 ± 0.3 µmol/L) in Group NC, with a gradual decrease thereafter (Figure 3A). BAC in the positive control group treated with Condition^®^ (Group PC) 1 h after ethanol administration was 80.8% (8.25 ± 0.66 µmol/L) of the level in Group NC, but the rate of decrease was similar. However, BAC in ethanol-loaded rats pretreated with 125–250 mg/kg b.w. of FSC (Groups FSC 125 and FSC 250) decreased rapidly after showing similar concentration levels as in Group PC 1 h after ethanol administration. BAC in the group treated with 500 mg/kg b.w. of FSC (52.7% [4.83 ± 1.0]) was significantly lower (47.3%) than that in Group NC. The final BACs 5 h after administration ranged from 1.14 ± 0.1 to 1.59 ± 0.3 µmol/L, with no significant difference between the groups. Furthermore, to measure the exposure that integrates concentration across time, the area under the concentration–time curve (AUC) was evaluated (Figure 3C). Consistently, the AUC of the group treated with FSC (125–500 mg/kg b.w.) was significantly (*p* < 0.05) lower than that in Group NC (61.5%) and similar to that in Group PC. The low BAC and AUC of the FSC pretreated groups, compared with Group NC, indicated that the relative bioavailability of alcohol after alcohol consumption was low.

The blood acetaldehyde concentrations and AUC were also determined in this study (Figure 3B,D). The blood acetaldehyde concentrations gradually increased after alcohol loading in rats. The blood acetaldehyde concentrations in groups pretreated with FSC (3.2 ± 0.85 to 3.8 ± 0.1 µmol/L) and Group PC (3.5 ± 0.1 µmol/L) 1 h after administration were lower than that in Group NC (4.8 ± 0.6 µmol/L). The AUC of acetaldehyde of the groups pretreated with FSC (250 and 500 mg/kg b.w.) was significantly (*p* < 0.05) lower (23.4–30.9%) than that in Group NC.

#### 3.2.2. Effect of FSC on Alcohol-Metabolizing Enzymes of Liver

**Figure 4:**
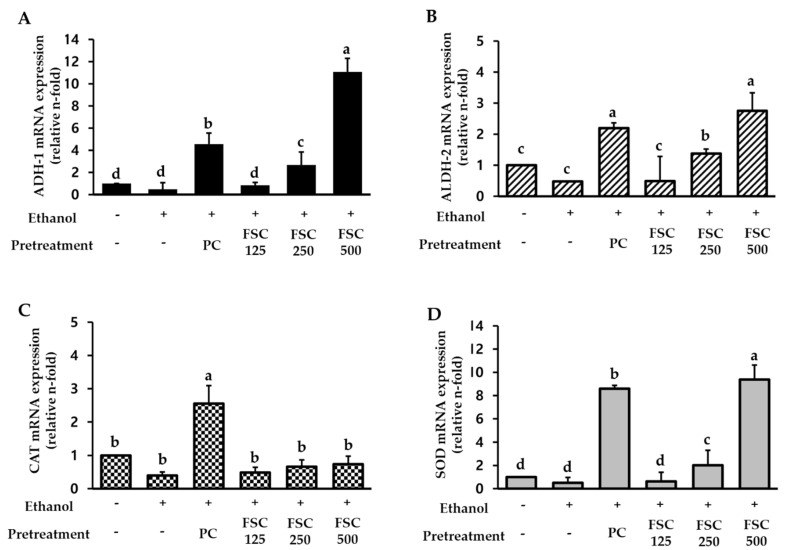
The effect of FSC on mRNA expression of alcohol metabolism-associated enzyme (**A**) and antioxidant enzyme (**B**,**C**) in rat livers after acute alcohol consumption. Livers were collected 5 h after ethanol administration. The relative mRNA expression levels of ADH-1, ALDH-2, CAT, and (**D**) SOD associated with alcohol metabolism or antioxidant activity were determined by RT-PCR. Data represent the mean ± SEM (n = 6). Bars with different letters show significant differences between groups (*p* < 0.05) determined by analysis of variance and Tukey’s multiple comparison test.

To elucidate the mechanisms underlying the reduction in serum alcohol and acetaldehyde concentrations by FSC in the ethanol-loaded rats, livers were extracted 5 h after ethanol administration. The gene expression levels of ADH-1, ALDH-2, and CAT in the extracted livers were evaluated by quantitative RT-PCR. Pretreatment with FSC (125–500 mg/kg b.w.) induced hepatic ADH-1 and ALDH-2 gene expression, but not CAT gene expression, in a dose-dependent manner (Figure 4A–C). There was a positive correlation between the reduction in serum alcohol and acetaldehyde concentrations. In particular, hepatic ADH-1 and ALDH-2 were significantly upregulated in rats pretreated with FSC (250 or 500 mg/kg b.w.) compared with that in Group NC.

The gene expression of SOD, which is an antioxidant enzyme in the liver, was also evaluated. FSC induced a dose-dependent increase in hepatic SOD gene expression (Figure 4D) in ethanol-loaded rats, and the increase was similar to that in Group PC.

### 3.3. Effect of FSC on Chronic Alcohol-Induced Hepatic Damage

#### 3.3.1. Effect of FSC on Serum Biochemical Parameters

Serum ALP (1364.7 ± 9.4), ALT (169.3 ± 4.9), and AST (47.3 ± 1.4) levels were significantly higher in Group NC than in Group PC (Table 1). ALT and AST levels in the groups pretreated with 500 mg/kg b.w. of FSC (Group FSC 500) decreased to ~34.5 and ~39.3%, respectively, compared with levels in untreated animals; however, AST level also decreased significantly (*p* < 0.05). There was no difference in the level of ALB between groups.

#### 3.3.2. Histopathology

**Figure 5:**
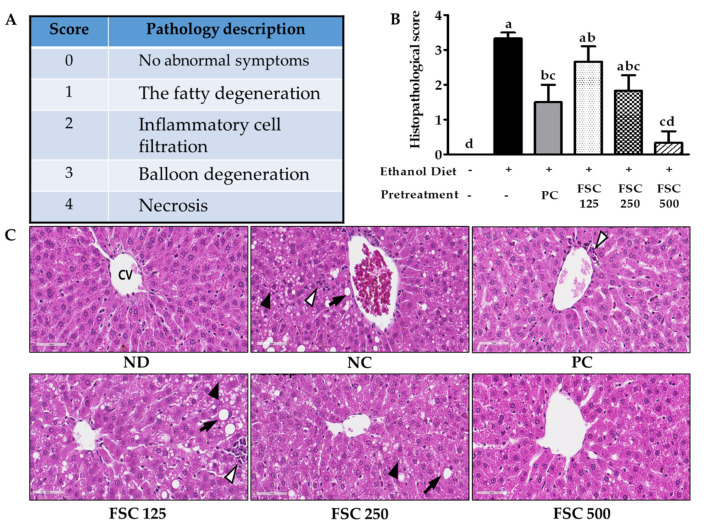
Liver histopathological scoring (**A**), effects of FSC on the histopathological score (**B**), and representative images of the liver histology (**C**). Group ND, normal diet; Group NC, ethanol diet only (negative control); Group PC, HDE + ethanol diet (positive control); Group FSC 125, 125 mg/kg b.w. FSC + ethanol diet; Group FSC 250, 250 mg/kg b.w. FSC + ethanol diet; Group FSC 500, 500 mg/kg b.w. FSC + ethanol diet. Data represent the mean ± SEM (n = 6). Bars with different letters show significant differences between groups (*p* < 0.05) determined by analysis of variance and Tukey’s multiple comparison test. The hepatocytes were analyzed to determine necrosis, fatty change, hepatocyte ballooning, and inflammatory cell infiltration. Tissues were fixed in formalin and stained with hematoxylin and eosin (200×): black arrows, macrovesicular droplets; arrowheads, microvesicular droplets; white arrows, inflammatory cell infiltration; CV, central vein.

The protective effect of FSC on chronic liver injury caused by alcohol consumption was determined based on the degree of pathological hepatic lesions (scores 0–4) (Figure 5A). The control group livers (Group ND) scored a ‘‘0,’’ having no pathological changes (Figure 5B,C).

The liver histopathological scores for the ethanol-treated group (Group NC) were 3.33 ± 0.17 as fatty change characterized by macrovesicular (large fat droplets per hepatocyte and lateral displacement of the nucleus) or microvesicular (many small fat droplets per hepatocyte) and inflammatory cell infiltration without balloon degeneration and necrosis of the hepatic cells. However, treatment with HDE at 250 mg/kg (Group PC) and FSC at 125, 250, or 500 mg/kg (Groups FSC 125, FSC 250, or FSC 500, respectively) significantly reduced most hepatic lesions caused by ethanol observed based on lesser fatty change and inflammatory cell infiltration. The scores for FSC-treated groups were 2.67 ± 0.44, 1.83 ± 0.44, and 0.33 ± 0.33, respectively. In particular, the score in Group FSC 500 was 5-fold lower than that in Group PC (1.5 ± 0.5) but similar to that in Group ND. These findings indicated that FSC improved alcohol-induced liver injury.

#### 3.3.3. Effect of FSC on the Expression of Lipid Metabolism-Related Genes in Rat Liver

**Figure 6:**
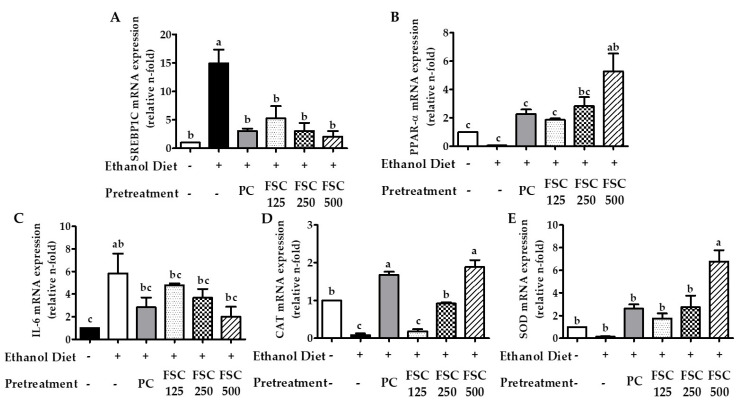
Effect of FSC on the mRNA expression of lipid metabolism-associated enzymes (**A**,**B**), inflammatory cytokine (**C**), and antioxidant enzyme (**D**,**E**) in rats with chronic alcohol consumption. Group ND, water (normal diet); Group NC, ethanol diet only (negative control); Group PC, HDE + ethanol diet (positive control); Group FSC 125, 125 mg/kg b.w. FSC + ethanol diet; Group FSC 250, 250 mg/kg b.w. FSC + ethanol diet; Group FSC 500, 500 mg/kg b.w. FSC + ethanol diet. The relative mRNA expression levels were determined by RT-PCR. Data represent the mean ± SEM (n = 6). Bars with different letters show significant differences between groups (*p* < 0.05) determined by analysis of variance and Tukey’s multiple comparison test.

The mechanism underlying the protective effect of FSC on the alcohol-induced fatty liver was further elucidated through quantitative RT-PCR. SREBP1C plays an important role in the pathophysiology of alcoholic hepatic steatosis. In the ethanol-fed rats pretreated with FSC, SREBP1C expression was significantly reduced (Figure 6A) (*p* < 0.05). The expression of PPAR-α (Figure 6B), which is associated with the oxidation of hepatic lipids, was also significantly decreased (63%) in the ethanol-fed rats (Group NC). The gene expression of PPAR-α increased in a dose-dependent manner in the ethanol-fed rats with FSC pretreatment (FSC 125, FSC 250, and FSC 500) compared with that in the ethanol-fed rats without pretreatment (Group NC). These results indicate that FSC may inhibit lipid accumulation in the liver after alcohol consumption.

#### 3.3.4. Effect of FSC on the Gene Expression of Anti-Inflammatory and Antioxidant Enzymes

IL-6 is a typical pro-inflammatory cytokine, and its gene expression was decreased in the ethanol-fed rats pretreated with FSC, with a 58.4% reduction in the group treated with 500 mg/kg b.w of FSC or 250 mg/kg b.w of HDE (Figure 6C). These results were consistent with histopathological observations and indicated that FSC could potentially protect against chronic alcohol-induced liver injury. The gene expression levels of hepatic CAT and SOD increased dose-dependently in the ethanol-fed rats pretreated with FSC (Figure 6D,E). The levels of CAT and SOD in Group FSC 500 were 4.9-fold and 19.8-fold, respectively, higher than those in Group NC and similar to those in Group PC. These findings suggest that an increase in CAT and SOD gene expression by FSC prevents alcohol metabolism and oxidative damage of ethanol consumption.

### 3.4. In Vivo Acute Oral Toxicity

The similarities in the number and type of detoxifying enzymes between rats and humans make rat models suitable for assessing the acute oral toxicity of FSC [24]. Bolus 2000 mg/kg b.w. of FSC was administered to female rats, and the rats were observed over 14 days. No clinical signs or deaths were observed in all animals after 2000 mg/kg b.w. oral dosing. Moreover, there was no significant difference among the bodyweight of both (control and FSC-treated) groups (Supplementary Tables S1–S4). Conducting the same study in all FSC-treated animals, no clinical signs or deaths were observed. Therefore, the acute toxic class method, following the flow chart of LD50 cut-off, confirmed FSC as a category 5 substance in the Globally Harmonized System of Classification and Labeling of Chemicals (GHS).

## 4. Discussion

Fermentation results in increased antioxidant activity of an extract due to the increased number of phenolic compounds and flavonoids as a direct result of microbial hydrolysis [27]. GABA is widely used as a food supplement [2]. GABA was abundantly present in the FSC used in this study. Our results demonstrated that GABA-enriched FSC extract enhances alcohol metabolism and thereby reduces alcohol-mediated liver injury. BAC, which reflects the effects of alcohol on various tissues, depends on the absorption, distribution, metabolism, and excretion of alcohol from the body after ingestion [28]. The rate of BAC elevation is affected by the first-pass metabolism associated with major alcohol-metabolizing enzymes such as ADH-1 and ALDH-2. Alcohol metabolism produces cytotoxic aldehyde via ALD-1 regulation and this aldehyde, by the regulation of ALDH-2 enzyme, is oxidized into acetate followed by ROS production. Recent studies have reported various plant extracts that enhance alcohol metabolism in rats [17,21]. In the present study, we observed that FSC improved hangover by stimulating hepatic alcohol metabolism. FSC pretreatment effectively decreased BAC and blood acetaldehyde concentration by inducing ADH-1 and ALDH-2 expression.

Alcohol consumption reduces SOD activity in some of the major organs and in the serum of rats. CAT acts as an antioxidant enzyme and protects against the deleterious effects of free radicals, but alcohol abuse also significantly reduces its activity [29]. Similar to previous observations, FSC increased SOD and CAT activities in rats. FSC pretreatment has been shown to increase cellular antioxidant capacity in alcohol-loaded rats by inducing the gene expression of CAT and SOD.

Chronic alcohol consumption and alcohol metabolism are strongly linked to several pathological consequences and tissue damage [6,30]. Therefore, in this study, the hepatoprotective effect of FSC was further evaluated in rats with chronic alcohol consumption. Serum ALT and AST have been most commonly used as laboratory parameters to assess liver function [31]. Administration of FSC maintained liver cell membrane stability by attenuating ethanol-induced liver damage, as demonstrated by a substantial reduction in serum ALP, ALT, and AST activities.

The accumulation of fat in the liver, which results in steatosis, occurs as a result of chronic alcohol consumption [32]. Alcohol ingestion induces fatty acid synthesis in the liver by increasing the expression of SREBP1c at both the gene and protein levels, which regulates the proteins involved in lipid synthesis by activating ATP citrate lyase and fatty acid synthase [33]. In contrast, PPARα inhibits proteins involved in fatty acid oxidation through substernal activation of acetyl-CoA carboxylase and carnitine palmitoyltransferase [34]. FSC attenuated alcohol-induced fat accumulation in the liver by downregulating SREBP1c and upregulating PPARα. This agreed with our histopathological observations showing a slight fatty change in the group pretreated with FSC.

IL-6 is a known important pro-inflammatory cytokine involved in hepatocyte injury due to chronic alcohol consumption [35]. FSC exhibits anti-inflammatory activity in chronic alcohol-induced liver damage by downregulating IL-6 gene expression. FSC administration did not markedly influence CAT oxidation, which is considered a minor pathway of alcohol oxidation [25], in rats administered a single alcohol load, whereas it increased CAT activity in chronic alcohol-treated rats. This indicated that FSC pretreatment might improve alcohol metabolism, preventing liver damage. In addition, FSC exhibited a strong antioxidant activity through induction of hepatic SOD gene expression in rats with not only single ethanol consumption but also with chronic alcohol consumption.

*S. china* is reported to contain phenolic compounds such as flavonoids and phenylpropanoids, including 7-O-β-D-glucoside, kaempferol, and 3-hydroxy-benzoic acid with various biological activities, including anticancer [14], anti-inflammatory [36], and antioxidant activities [37]. In addition, oxyresveratrol, resveratrol [38], and steroidal saponins [39] have anti-inflammatory and antioxidant properties. At present, it is not clear which single or multiple compounds present in FSC are responsible for its hepatoprotective activity. The reported phenolic compounds in the extract of *S. china* may play a role in the hepatoprotective effect on alcohol-loaded rats. A study has also reported that GABA can protect against the cytotoxic effect of ethanol due to its ability to maintain the level of polyamines in the cell [40]. Thus, physiologically active compounds and GABA in FSC may synergistically protect against alcohol-induced liver damage. However, the specific physiological compounds for the hepatoprotective activity in FSC should be evaluated in future studies.

## 5. Conclusions

Our study demonstrated that GABA-enriched FSC protects the liver from alcohol-induced damage by increasing not only alcohol metabolism through regulation of ADH-1 and ALDH-2 enzymes but also by increasing the tissue antioxidant potential through the regulation of SOD and CAT expression. Moreover, FSC significantly protected the liver cells and reduced the severity of liver lesions caused by alcohol intoxication. The underlying mechanisms include upregulation or downregulation of the transcription factors involved in lipid metabolism, oxidation, and inflammation (PPAR-α, SOD, CAT, SREBP-1c, and IL-6). These results suggest that FSC could be used as a functional food ingredient for attenuating hangovers after excessive alcohol consumption, as well as for protecting against alcohol-induced hepatic damage. Optimization of the fermentation process would greatly affect the duration and intensity of action of FSC. Therefore, the effect of physical parameters of the fermentation procedure (temperature, duration, and number of cycles), the effect of various probiotics and their combinations on the polyphenolic content, and the effects of alcohol overdose, fatty liver, and cirrhotic liver in animal models are future directions of this study. For this purpose, further detailed studies are required to develop FSC into a product with good clinical efficacy.

## Figures and Tables

**Table 1:** Effects of FSC on liver biomarkers of alcohol-induced liver injury in rats.

Treatment (per kg b.w.)	ALP (U/L)	ALT (U/L)	AST (U/L)	ALB (U/L)
**ND**	857.0 ± 7.8 ^b^	107.3 ± 3.8 ^b^	26.3 ± 5.5 ^c^	4.0 ± 0.1 ^a^
**NC**	1364.7 ± 9.4 ^a^	169.3 ± 4.9 ^a^	47.3 ± 1.4 ^a^	4.0 ± 0.2 ^a^
**PC**	780.8 ± 4.8 ^b^	102.8 ± 2.1 ^b^	36.5 ± 4.7 ^b^	3.7 ± 0.1 ^a^
**FSC 125**	1311.3 ± 4.1 ^a^	99.0 ± 9.4 ^b^	36.3 ± 4.4 ^b^	3.6 ± 0.2 ^a^
**FSC 250**	1018.0 ± 7.4 ^c^	99.0 ± 6.1 ^b^	37.3 ± 4.4 ^ab^	3.5 ± 0.1 ^a^
**FSC 500**	985.0 ± 9.7 ^b,c^	100.5 ± 9.6 ^b^	28.7 ± 1.2 ^c^	3.9 ± 0.1 ^a^

The rats were treated with FSC (125, 250, or 500 mg/kg b.w.) once daily with or without Lieber-DeCarli ethanol (6.7%) diet for 28 consecutive days. Hepatic injury was determined by quantifying the serum activities of alkaline phosphatase (ALP), alanine aminotransferase (ALT), aspartate aminotransferase (AST), and serum albumin (ALB). Data represent the mean ± SEM (n = 6). Bars with different letters show significant differences between groups (*p* < 0.05) determined by analysis of variance and Tukey’s multiple comparison test.

## Data Availability

Data supporting the present study are available from the corresponding author upon request.

## Supplementary Materials

The following are available online at https://www.mdpi.com/article/10.3390/foods10102381/s1, Table S1: gross findings in rats treated with FSC, Table S2: individual clinical signs induced by the treatment of FSC, Table S3: changes in bodyweight after oral administration with/without FSC, Table S4: mortality in rats after single oral administration with/without FSC.
