# Supplementary material for: Ethanol-Induced Hepatotoxicity and Alcohol Metabolism Regulation by GABA-Enriched Fermented Smilax china Root Extract in Rats
Source: Foods. 2021 Oct 8;10(10):2381. doi: 10.3390/foods10102381 (PMC8535858; doi:10.3390/foods10102381)
Supplement: Supplementary file 1 [file foods-10-02381-s001.zip › foods-1368475-supplementary.pdf]

Supplementary Data

Table S1. Gross findings in rats treated with FSC.

Group	Sex	Dose	Animal Number	Day of Death	Gross findings
I	Female	Non-treated	1	Day 15	NGF[a]
			2	Day 15	NGF
			3	Day 15	NGF
			4	Day 15	NGF
			5	Day 15	NGF
II	Female	2000 mg/kg b.w.	1	Day 15	NGF
			2	Day 15	NGF
			3	Day 15	NGF
			4	Day 15	NGF
			5	Day 15	NGF

[a]No gross findings (NGF): internal and external gross findings were not observed.

Table S2. Individual clinical signs induced by treatment with FSC.

Group	Sex	Dose	Animal Number	0.5 hour	1 hour	2 hour	3 hour	4 hour	Day 1	Day 2–14
I	Female	Non-treated	1	NCS[a]	NCS	NCS	NCS	NCS	NCS	NCS
			2	NCS	NCS	NCS	NCS	NCS	NCS	NCS
			3	NCS	NCS	NCS	NCS	NCS	NCS	NCS
			4	NCS	NCS	NCS	NCS	NCS	NCS	NCS
			5	NCS	NCS	NCS	NCS	NCS	NCS	NCS
II	Female	2000 mg/kg b.w.	1	NCS	NCS	NCS	NCS	NCS	NCS	NCS
			2	NCS	NCS	NCS	NCS	NCS	NCS	NCS
			3	NCS	NCS	NCS	NCS	NCS	NCS	NCS
			4	NCS	NCS	NCS	NCS	NCS	NCS	NCS
			5	NCS	NCS	NCS	NCS	NCS	NCS	NCS

[a]No Clinical signs (NCS): internal and external gross findings were not observed

Table S3. Changes in bodyweight after oral administration with and without FSC.

Groups	Days after treatments							
	0	1	3	5	7	10	12	14
Control (g)	321.0 ± 0.0	341.0 ± 0.0	371.0 ± 0.0	384.0 ± 0.0	391.0 ± 0.0	412.0 ± 0.0	432.0 ± 0.0	435.0 ± 0.0
FSC (g)	353.0 ± 25.9	366.4 ± 11.3	380.0 ± 15.1	389.4 ± 16.7	386.6 ± 13.2	397.0 ± 10.4	421.8 ± 11.9	429.8 ± 12.8

Data represent the means ± SEM (n = 6). Bars with different letters show significant differences between groups ($p < 0.05$) determined by T-test.

Table S4. Mortality in rats after a single oral administration with and without FSC.

Groups	Days after dosing															Final Mortality
	0	1	2	3	4	5	6	7	8	9	10	11	12	13	14	
Control	0	0	0	0	0	0	0	0	0	0	0	0	0	0	0	0/6
FSC	0	0	0	0	0	0	0	0	0	0	0	0	0	0	0	0/6
